# Classic Kaposi sarcoma in a patient of Miao ethnicity followed up for 7 years: a case report

**DOI:** 10.1186/s13256-021-02777-7

**Published:** 2021-04-19

**Authors:** Jing Zhou, Xiaoping Shen, Xiaodong Wang, Kun Xiao, Yu Cao, Yanping Jiang

**Affiliations:** 1 Department of Dermatology, The Affiliated Hospital, Guizhou Medical University, Beijing Road 4, Yunyan District, Guiyang, China; 2 Department of Dermatology, The Affiliated Hospital, XingJiang Medical University, Urumqi, China; 3 Department of Radiology, Guiyang Third People’s Hospital, Guiyang, China; 4 Centre of Expertise in Mycology of RadboudUMC / CWZ, Nijmegen, The Netherlands

**Keywords:** Human herpesvirus 8, Kaposi sarcoma, Tomography, Ethnic groups, Genotype

## Abstract

**Background:**

Classic Kaposi sarcoma (CKS) is a vascular sarcoma associated with human herpesvirus 8 (HHV-8), which is known to be more common in Mediterranean elderly men and is characterized by indolent clinical behavior. Xinjiang province in China is considered an endemic region for Kaposi’s sarcoma-associated herpesvirus (KSHV), with higher incidence among adults of Kazak and Uyghur ethnicities. Cases of CKS are rarely reported in inland China. Here, we followed a case of CKS for 7 years in a patient of Miao ethnic background in southwestern China.

**Case presentation:**

A 63-year-old Miao (southwestern China) man was initially diagnosed with CKS in 2010, having a history of limb lesions for 37 years, with left eyelid and binaural lesions for 9 years. He did not have sexual contact with men and was human immunodeficiency virus (HIV)-negative. Due to his lumbago and fever, spinal tuberculosis in the lumbar vertebra was highly suspected after computed tomography (CT) scan. However, diagnostic antituberculosis treatment for 4 weeks failed. The patient was followed up in 2016, when the rash was recovering as the systemic symptoms improved. A new CT was performed, which showed a partial response despite the absence of any medical treatment. The open reading frame (ORF)-K1 of KSHV from skin tissue of the foot was amplified and sequenced, and K1 belonged to subtype A. This genotype is consistent with the typical subtype present in Xinjiang.

**Conclusions:**

We describe spontaneous partial regression of CKS in a patient of Miao ethnicity in inland China. Our sample may represent an unknown, novel genotype. Surveillance and regulating the immune state may represent a valuable approach for this rare disease.

## Background

Human herpesvirus 8 (HHV-8), also known as Kaposi's sarcoma-associated herpesvirus (KSHV), is commonly associated with Kaposi's sarcoma (KS). There are four different epidemiological forms of the disease—classic, endemic, iatrogenic and acquired immunodeficiency syndrome (AIDS)-associated. Classic Kaposi sarcoma (CKS) is a low-grade malignant tumor derived from the vascular endothelial cells, and is more common in Mediterranean elderly men aged 50 to 80 years [1]. It is characterized by indolent clinical behavior and often causes skin damage at the end of the lower extremities, with less visceral damage [2]. The disease is associated with ethnic and geographic background [3]. In China, CKS cases are rare and have been reported in the Xinjiang region and Taiwan [4]. There are reports that the prevalence of KSHV and incidence of CKS are high among people of Uygur ethnicity in Xinjiang [3]. Here, we followed a CKS case for 7 years in a patient of Miao ethnic background in southwestern China.

## Case presentation

**Fig. 1:**
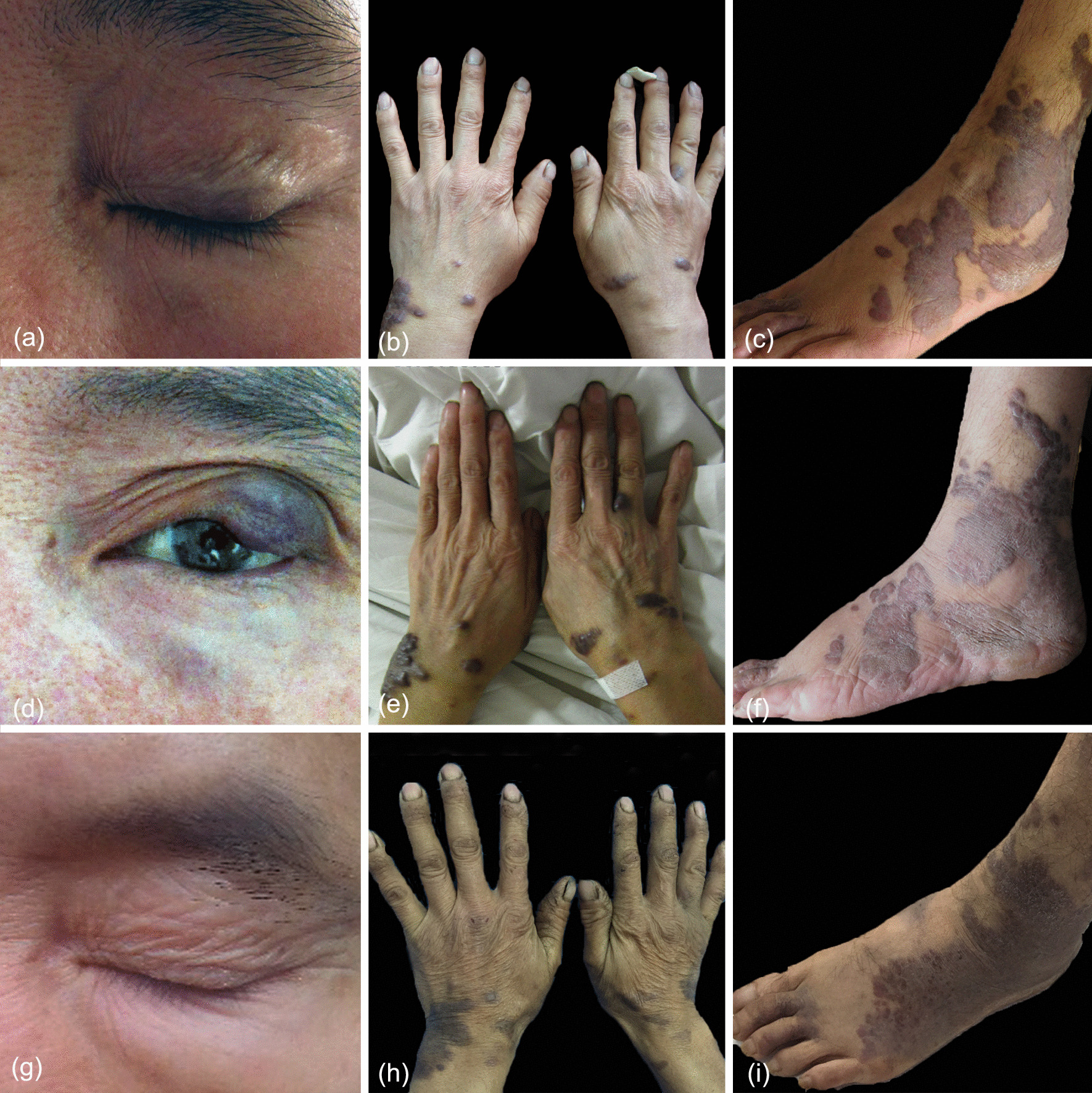
Dynamic changes in the rash in this patient. Multiple blue and purplish red nodules varying in size affecting the upper left eyelid, the bilateral auricle, limbs, hands and forefoot in April 2010 (**a**, **b**, **c**). The rash increases with lower back pain in Dec 2010 (**d**, **e**, **f**). Clinical improvement with reduction in dimension/absence of nodules in July 2016 (**g**, **h**, **i**)

**Fig. 2:**
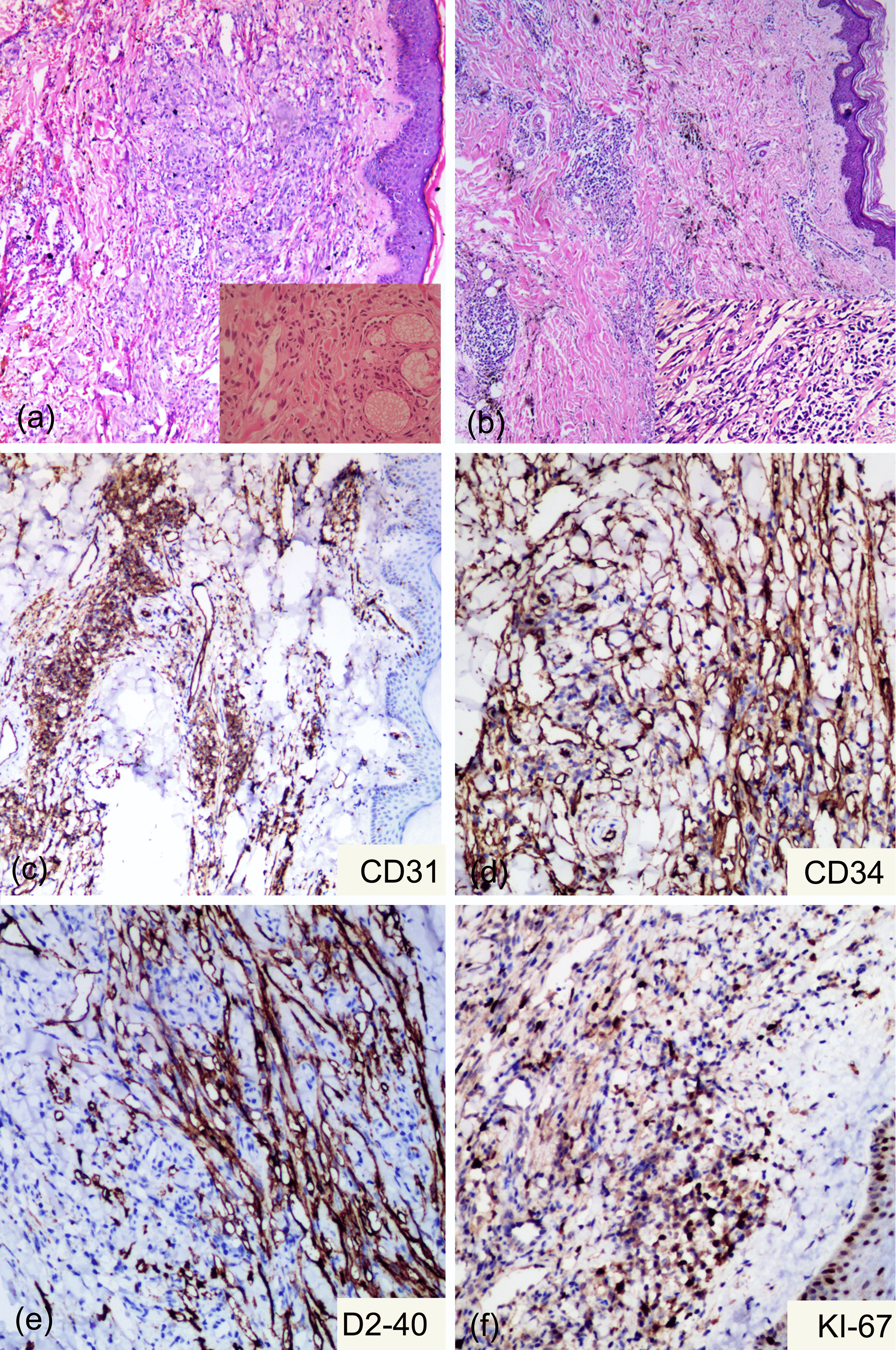
Dynamic histopathological changes in the lesion of the right foot in this patient. Haematoxylin and eosin staining (×100 original magnification photomicrograph) showing proliferation of fusiform cells and endothelial cells, with extravasation of red blood cells and intervening slit-like spaces in April 2010 (**a**) and decreased cell proliferation and increased deposition of hemosiderin in July 2016 (**b**). Histology and immunohistochemistry show positivity for CD31, CD34, D2-40 and Ki67 (**c**, **d**, **e**, **f**)

A 63-year-old Miao (southwestern China) man was initially diagnosed with CKS in April 2010, with a history of limb lesions for 37 years and left eyelid and binaural lesions for 9 years. The patient was a farmer who had been living in Zhijin County of Guizhou Province for many years. He had been abstaining from alcohol for 10 years and continued smoking. His test for human immunodeficiency virus (HIV) was negative, with no associated immunodeficiency and no special family or psychosocial history. He denied having sexual contact with men. Routine laboratory tests were all normal. The vital signs were found to be stable. Examination of the skin showed multiple blue and purplish red patches and nodules varying in size from 1 to 4 cm, affecting the upper left eyelid, the bilateral auricle, limbs, hands and forefoot (Fig. 1a–c). Pathological biopsy of the right foot skin tissue suggested proliferation of fusiform cells and endothelial cells, with extravasation of red blood cells and intervening slit-like spaces (Fig. 2a).

**Fig. 3:**
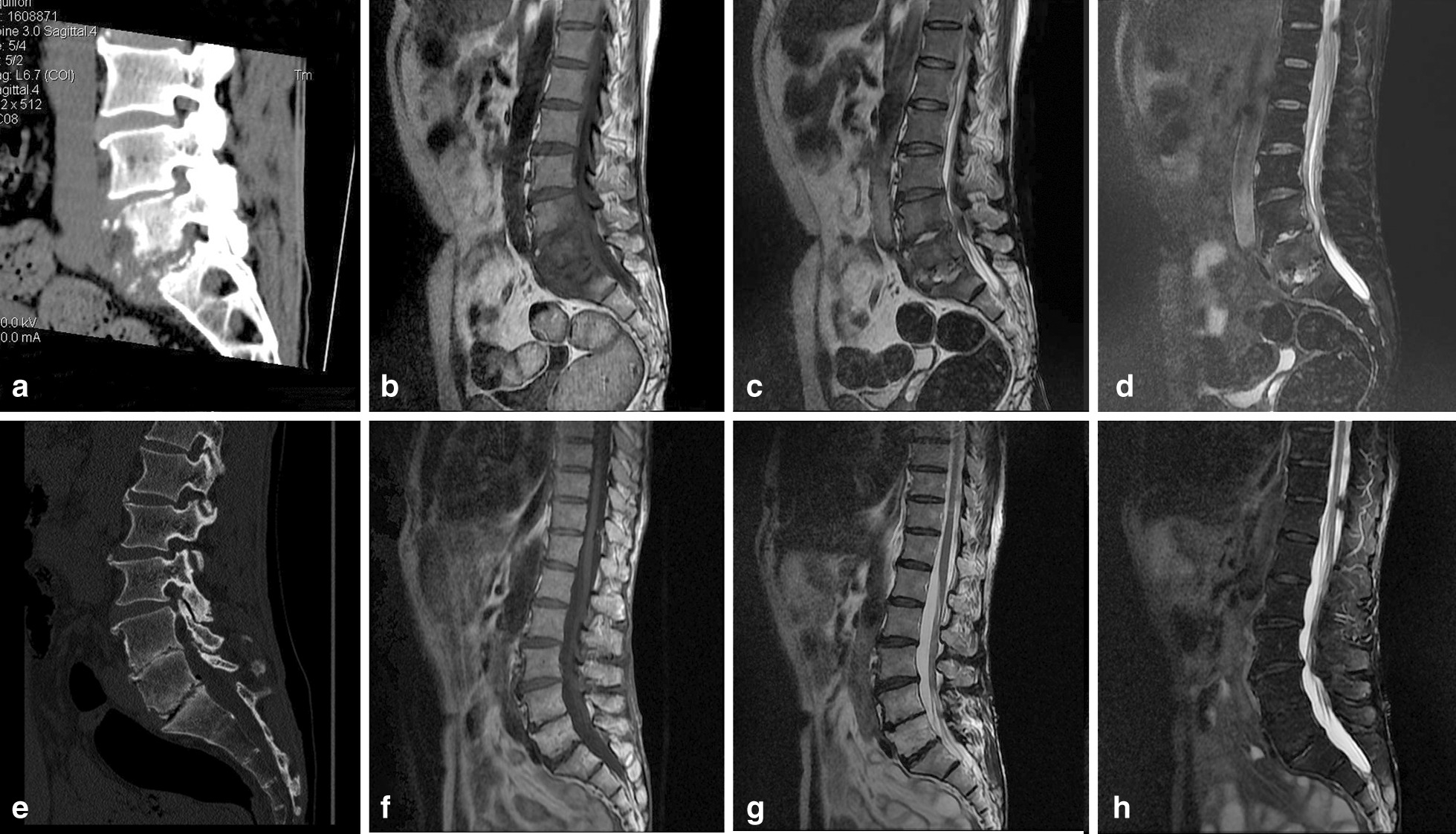
Dynamic changes in the lumbar vertebra on computed tomography (CT) scan. Bone destruction at the upper and lower edges of the lumbar vertebra and the superior border of the sacrum were observed in Dec 2010 (**a**, **b**, **c**, **d**). No obvious bone destruction and disappearance of soft tissue swelling in July 2016 (**e**, **f**, **g**, **h**)

Several months later, he presented with lumbago and fever, and spinal tuberculosis in the lumbar vertebra was highly suspected after computed tomography scan (CT) in December 2010. Bone destruction at the upper and lower edges of the lumbar vertebra and the superior border of the sacrum was reported (Fig. 3a–d). Physical examination showed stiffness in the physiological curvature of the spine and limited movement, along with decreased pain in both lower extremities, positive straight leg raising test, positive strength test of both lower extremities, positive for bilateral Thomas sign, positive for strong reflexes in both knees, negative for reflexes in left and right ankle joints, and positive for Babinski sign. Erythrocyte sedimentation rate (ESR) was 96 mm/hour (normal range 0–15 mm/hour), and C-reactive protein (CRP) was 209.40 mg/L (normal range 0.00–8.00 mg/L). Diagnostic treatment with isoniazid (300 mg/day) and rifampicin (450 mg/day) for 4 weeks showed poor efficacy, and skin lesions increased with fever (Fig. 1d–f). He refused to continue taking antituberculosis drugs and returned home to recuperate. The patient's systemic symptoms resolved spontaneously after 3 months.

**Fig. 4:**
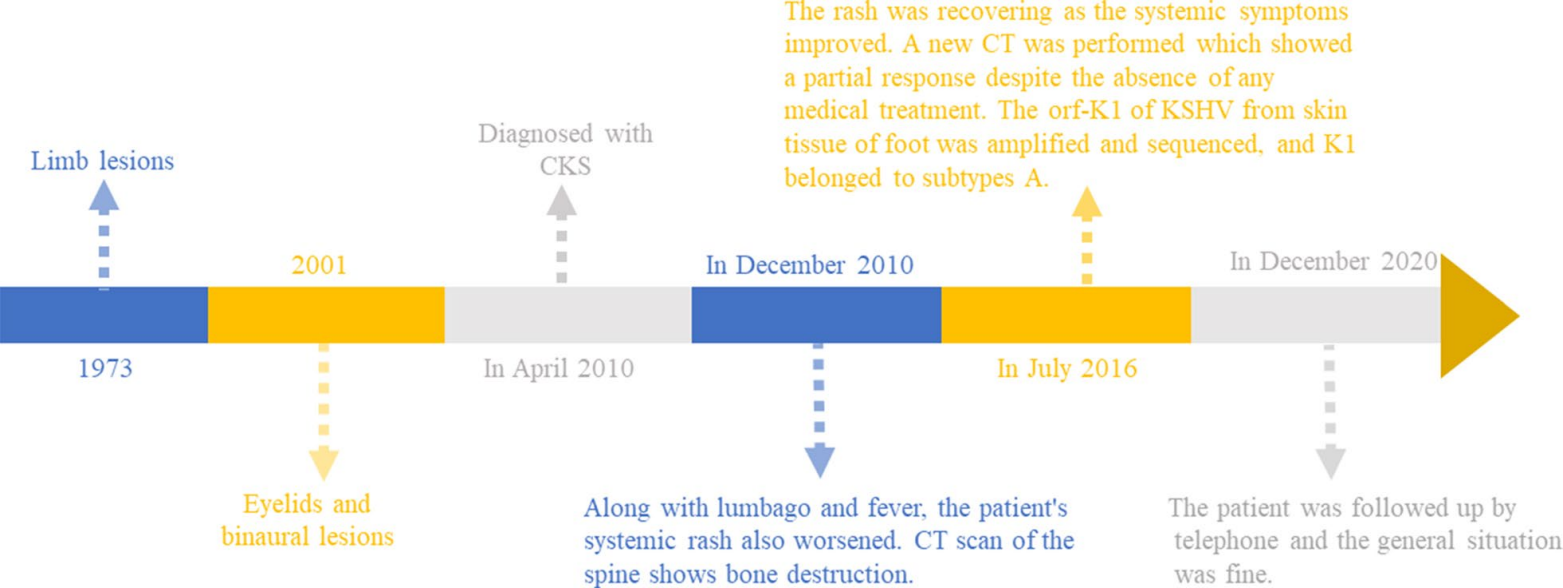
A timeline of the patient's disease progression

The patient was followed up again in July 2016, when a new CT was performed, and the image showed a partial response despite the absence of any medical treatment (Fig. 3e–h). The rash was recovering as the systemic symptoms improved (Fig. 1g–i). Laboratory investigations showed no abnormality with the exception of a slight elevation of the blood immunoglobulin G (IgG) to 17.6 g/L (normal range 11.51–14.22 g/L). The pathological examination of the skin lesions on the right foot again showed that the fissure-like blood vessels, erythrocyte extravasation and spindle cells were reduced, and the deposition of hemosiderin was increased (Fig. 2b). Immunohistochemistry test results were positive for CD31, CD34 and D2-40(+); Ki67 was 20% positive (Fig. 2c–f). A serology test for HHV-8 was negative. The open reading frame (ORF)-K1 of KSHV from skin tissue of the foot was amplified and sequenced [4]. The sequence of our sample (JYP 2016) was compared to the National Center for Biotechnology Information (NCBI) GenBank database. Phylogenetic analysis was performed to confirm its identity and to infer its relationship with related species using the Randomized Axelerated Maximum Likelihood (RAxML) program and MrBayes version 3.1.2 Bayesian inference (BI) analysis, respectively [5]. Based on the data analysis, the sequence was inferred as belonging to subtype A, and may represent an unknown genotype (Fig. 4).

**Fig. 5:**
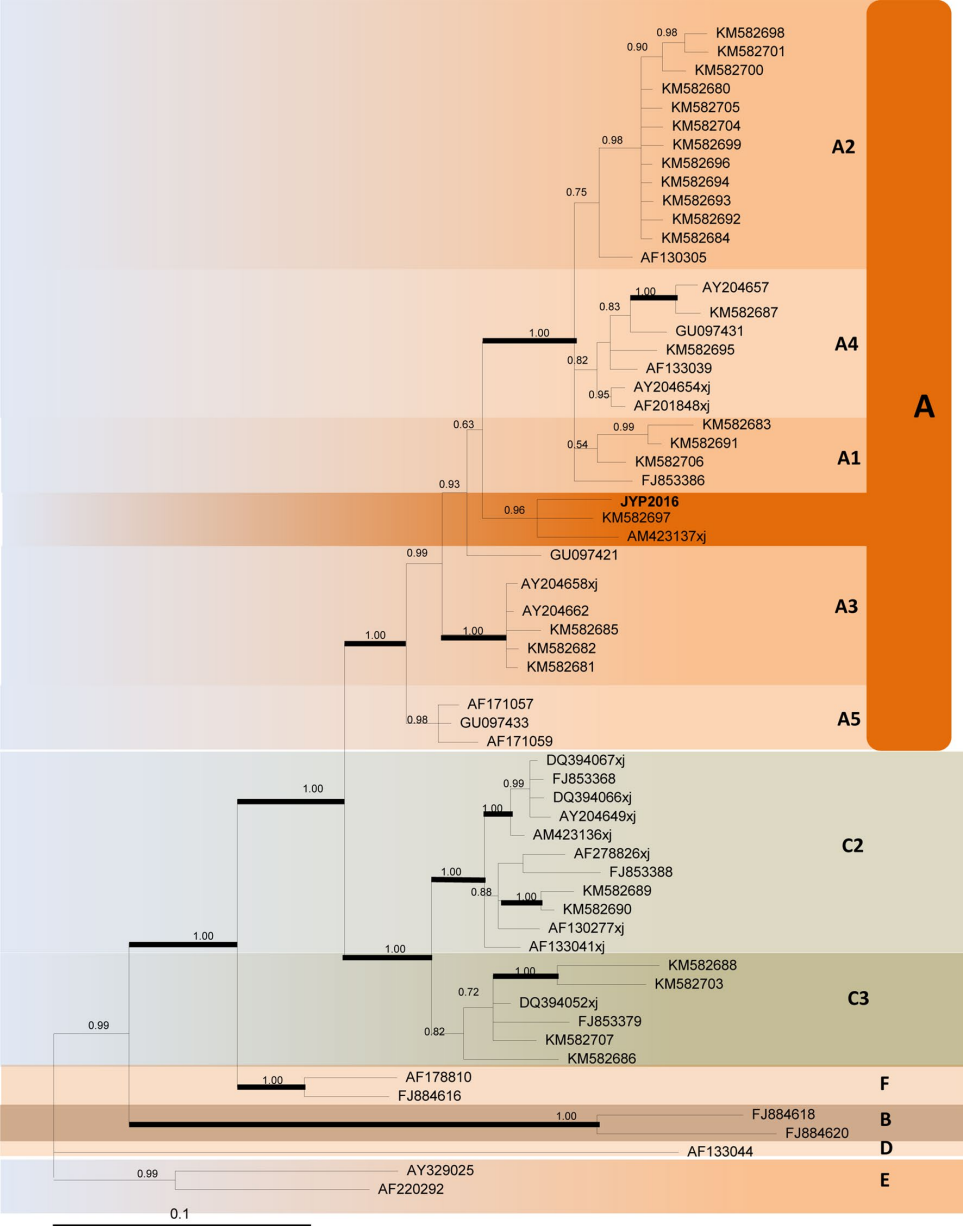
Consensus phylogram (50% majority rule) resulting from maximum likelihood and MrBayes analysis of the Kaposi's sarcoma-associated herpesvirus (KSHV) open reading frame (ORF)-K1 deoxyribonucleic acid (DNA) sequence alignment, with the confidence values of bootstrap analysis above branches (> 50%). Genotypes are individually labeled

The patient was followed up by telephone in December 2020, and the general situation was fine. The rash was slightly smaller in scope and lighter in color than before, accompanied by occasional itching, but no special discomfort was reported and no treatment was given. The patient is still being followed up. The timeline of his disease progression is show in Fig. 5.

## Discussion and conclusions

In this case, the diagnosis of CKS was confirmed based on clinical and pathological features and a positive HHV-8 deoxyribonucleic acid (DNA) test of the cutaneous lesion. To our knowledge, this case from Guizhou Province in southwestern China is the first reported CKS patient of Miao ethnic background in the world.

There is still no standard treatment for CKS; however, the community agrees that the management should focus on controlling the disease [6]. The prognosis and choice of treatment normally depend on the type of KS and the global assessment of the patient, in particular the patient's immune status [7]. The presence of herpesvirus infection and any concomitant changes in the balance of the immune system are well-recognized factors promoting the development of this disease [8–11]. Vincenzi *et al*. described the spontaneous partial regression of non-HIV (human immunodeficiency virus)-associated, non-drug-induced Kaposi's sarcoma [12], which provides a potential treatment strategy where close monitoring and regulation of immune status may be a valuable approach in specific cases.

In our patient, the severity of the rash was consistent with the general immune status. When the patient was diagnosed with spinal tuberculosis with low back pain, the rash had worsened all over the body. We were unable to verify the presence of tuberculosis due to the lack of evidence in pathology and etiology. We were also not sure whether CKS was responsible for the bone destruction, even though the absence of periosteum reaction and the cystic lesion of osteolytic bone did coincide with the imaging examination of bone destruction of CKS [13]. The follow-up examination showed that the bone destruction in the patient had improved without special treatment, and the rash tended to resolve. As a result, we assume that a fully competent immune system may play a role in the observed regression of the disease without chemotherapy-induced damage. However, future studies are needed to verify the reliability of this hypothesis, and additional data are required to support close surveillance as a management strategy in this rare disease.

HHV-8 has oncogenic potential, since multiple viral genes could regulate the pathways controlling the switch between latent and lytic replication [14]. Seven subtypes of KSHV (A, B, C, D, E, F and Z) have been identified and associated with the geographic and ethnic background of patients [4, 14]. Isaacs *et al*. [15] confirmed that subtypes A5 and B are predominant in South Africa, and A5 may be associated with more extensive disease [15]. Xinjiang province is considered an endemic KSHV region in China, with higher incidence among adults of Kazak and Uyghur ethnicities. KSHV subtypes in the Xinjiang region include subtypes A and C, and subtypes A is predominant. Our case was identified as subtypes A, and may represent an unreported genotype. Typically, CKS has been predominantly found in elderly Uyghur men over 60 years of age in Xinjiang [3], but our case represents the first CKS patient of Miao ethnic background reported from inland China. Herpesviruses have been considered to co-evolve with their hosts throughout the evolutionary history of vertebrates. KSHV is an ancient human virus and spread with the migration of its human hosts [4]. Previous studies suggest that KSHV was introduced to the Xinjiang region along the Silk Road [4]. However, Miao people have long been isolated in the mountainous areas of Guizhou, which is by no means part of the Silk Road belt and region. Further studies should be launched to elucidate the transmission pathway, the epidemiological pattern and the clinical relevance of KSHV infection in inland China.

In conclusion, the special genetic background of this case and the follow-up observational strategy in this study may provide a new perspective for further understanding of this disease. We hypothesize that maintaining a normal immune system, rather than administering chemotherapeutic drugs that disrupt the body's immunity, may play an important role in the outcome of CKS.

## Data Availability

Data sharing is not applicable to this article, as no data sets were generated or analyzed during the current study.

## Abbreviations

CKS: Classic Kaposi sarcoma
HHV-8: Human herpesvirus 8
KSHV: Kaposi’s sarcoma-associated herpesvirus
CT: Computed tomography
ESR: Erythrocyte sedimentation rate
CRP: C-reactive protein
IgG: Immunoglobulin G

## References

[1] Chang Y, Cesarman E, Pessin MS, Lee F, Culpepper J, Knowles DM, Moore PS. Identification of herpesvirus-like DNA sequences in AIDS-associated Kaposi’s sarcoma. Science. 1994;266(5192):1865–9. 10.1126/science.7997879 7997879

[2] Mohanna S, Maco V, Bravo F, Gotuzzo E. Epidemiology and clinical characteristics of classic Kaposi’s sarcoma, seroprevalence, and variants of human herpesvirus 8 in South America: a critical review of an old disease. Int J Infect Dis. 2005;9(5):239–50. 10.1016/j.ijid.2005.02.004 16095940

[3] Zheng J, Yang Y, Cui M, Shu ZJ, Han LL, Liu ZQ, et al. Prevalence of Kaposi’s sarcoma-associated herpesvirus in Uygur and Han populations from the Urumqi and Kashgar regions of Xinjiang, China. Virol Sin. 2017;32(5):396–403. 10.1007/s12250-017-4049-9. PMC6704202 29116591

[4] Ouyang X, Zeng Y, Bishi Fu, Wang X, Chen W, Fang Y, et al. Genotypic analysis of Kaposi’s sarcoma-associated herpesvirus from patients with Kaposi’s sarcoma in Xinjiang, China. Viruses. 2014;6:4800–10. 10.3390/v6124800. PMC4276930 25431948

[5] Ronquist F, Huelsenbeck JP. MrBayes 3: Bayesian phylogenetic inference under mixed models. Bioinformatics. 2003;19:1572–4. 10.1093/bioinformatics/btg180 12912839

[6] Lebbe C, Garbe C, Stratigos AJ, Harwood C, Peris K, Marmol VD, et al. Diagnosis and treatment of Kaposi’s sarcoma: European consensus-based interdisciplinary guideline (EDF/EADO/EORTC). Eur J Cancer. 2019;114:117–27. 10.1016/j.ejca.2018.12.036. 31096150

[7] Cattelan A, Trevenzoli M, Aversa S. Recent advances in the treatment of AIDS-related Kaposi’s sarcoma. Am J Clin Dermatol. 2002;3(7):451–62. 10.2165/00128071-200203070-00002 12180893

[8] Douglas JL, Gustin JK, Dezube B, Pantanowitz JL, Moses AV. Kaposi’s sarcoma: a model of both malignancy and chronic inflammation. Panminerva Med. 2007;49(3):119–38. 17912148

[9] Brown EE, Whitby D, Vitale F, Marshall V, Mbisa G, Gamache C, et al. Virologic, hematologic, and immunologic risk factors for classic Kaposi sarcoma. Cancer. 2006;107(9):2282–90. 10.1002/cncr.22236 16998933

[10] Sung JC, Louie SG, Park SY. Kaposi’s sarcoma: advances in tumor biology and pharmacotherapy. Pharmacotherapy. 1997;17(4):670–83. 9250545

[11] Galleu A, Fozza C, Simula MP, Contini S, Virdis P, Corda G, et al. CD4+ and CD8+ T-Cell Skewness in classic Kaposi sarcoma. Neoplasia. 2012;14(6):487–94. 10.1596/neo.11646 PMC3394191 22787430

[12] Vincenzi B, D’Onofrio L, Frezza AM, Grasso RF, Fausti V, Santini D, et al. Classic Kaposi sarcoma: to treat or not to treat? BMC Res Notes. 2015;10(8):138. 10.1186/s13104-015-1076-1. PMC4395989 25889316

[13] Caponetti G, Dezube BJ, Restrepo CS, et al. Kaposi sarcoma of the musculoskeletal system: a review of 66 patients. Cancer. 2007;109(6):1040–52. 10.1002/cncr.22500 17265518

[14] Cordiali-Fei P, Trento E, Giovanetti M, Presti AL, Latini A, Giuliani M, et al. Analysis of the ORFK1 hypervariable regions reveal distinct HHV-8 clustering in Kaposi’s sarcoma and non-Kaposi’s cases. J Exp Clin Cancer Res. 2015;34:1. 10.1186/s13046-014-0119-0. PMC4311464 25592960

[15] Isaacs T, Abera AB, Muloiwa R, Katz AA, Todd G. Genetic diversity of HHV8 subtypes in South Africa: A5 subtype is associated with extensive disease in AIDS-KS. J Med Virol. 2016;88(2):292–303. 10.1002/jmv.24328. 26174882

